# The relationship between ultra-processed food intake and cardiometabolic risk factors in overweight and obese women: A cross-sectional study

**DOI:** 10.3389/fnut.2022.945591

**Published:** 2022-08-09

**Authors:** Dorsa Hosseininasab, Farideh Shiraseb, Sahar Noori, Shahin Jamili, Fatemeh Mazaheri-Eftekhar, Mahshid Dehghan, Alessandra da Silva, Josefina Bressan, Khadijeh Mirzaei

**Affiliations:** ^1^Department of Nutrition, Science and Research Branch, Islamic Azad University, Tehran, Iran; ^2^Department of Community Nutrition, School of Nutritional Sciences and Dietetics, Tehran University of Medical Sciences (TUMS), Tehran, Iran; ^3^Department of Surgery, Shahid Beheshti University of Medical Sciences, Tehran, Iran; ^4^Population Health Research Institute, McMaster University and Hamilton Health Sciences, Hamilton, ON, Canada; ^5^Department of Nutrition and Health, Universidade Federal de Viçosa, Viçosa, MG, Brazil

**Keywords:** ultra-processed food, cardiovascular diseases, obesity, overweight, cardiometabolic risk

## Abstract

**Background:**

Cardiovascular diseases (CVDs) are the leading cause of death globally. Based on recent studies, one of the factors that can have detrimental effects on CVD is the consumption of ultra-processed foods (UPFs). The current study investigated the relationship between UPF intake and cardiometabolic risk factors among Iranian women.

**Methods:**

The current cross-sectional study was conducted on 391 women aged 18–65 years with a body mass index (BMI) ≥ 25 kg/m^2^. Dietary intake was assessed using a 147-item food frequency questionnaire (FFQ). Anthropometric and biochemistry parameters were also collected. UPFs were identified using the NOVA classification.

**Results:**

In the present study, women had a mean (standard deviation) age of 36.67 (9.10) years and the mean BMI of 31.26 (4.29) kg/m^2^. According to our findings, there was a significant association between UPF consumption and transforming growth factor (TGF) (β: 0.101, 95% CI: 0.023, 0.180, *p* = 0.012), atherogenic coefficient (AC) (β: 0.011, 95% CI: 0.001, 0.032, *p* = 0.034), visceral fat level (VFL) (β: 0.006, 95% CI: −0.017, 0.029, *p* = 0.076), and the quantitative insulin sensitivity check index (QUICKI) (β: −3.775, 95%CI: 0.001, 0.001, *p* = 0.042).

**Conclusion:**

In conclusion, an increase in consumption of one gram of UPFs is associated with an increase in TGF, AC, and VFL but with a decrease in QUICKI. Despite this, further experimental studies are necessary to draw a more definite conclusion and disentangle the mechanisms by which UPFs may affect health.

## Introduction

Cardiovascular diseases (CVDs) are the leading cause of death globally; an estimated 17.9 million people died from CVDs in 2019, representing 32% of all global deaths (1). About 85% of the deaths were due to heart attack and stroke (1). According to previous studies conducted in 2016 and 2017, CVD has been the major cause of mortality in Iran, accounting for 46% of all deaths and 20–23% of the disease burden (2, 3).

The global consumption of ultra-processed foods (UPF) has risen exponentially. UPFs account for between 25 and 60% of total daily energy consumption, according to the Nationwide Food Surveys (4–14). According to the NOVA classification system, UPFs are defined as foods made up entirely or predominantly from unhealthy components containing higher levels of total fat, saturated fat, added sugar, energy density, and salt, and lower quantities of fiber and vitamin density (15). UPF packaging contains materials that come into contact with food, such as Bisphenol A, which, according to a meta-analysis of observational studies, may increase the risk of cardiometabolic disorders, even though prospective cohort studies are still limited (16, 17). Some studies reported that consumption of UPF is associated with adverse health outcomes, including CVDs, and obesity (18–20). Srour et al. reported a higher risk of CVD associated with the consumption of ultra-processed foods (21).

Given the high prevalence of CVDs in Iran, it is necessary to find dietary factors that may associate with the disease (22). The main objective of this study was to investigate the relationship between UPF intake and cardiometabolic risk factors among Iranian women, and the secondary objectives were to exhibit the association between UPF consumption, food groups, and demographic variables.

## Methods

### Study population

The research was conducted in Tehran, Iran, using a multi-stage cluster random sampling procedure on 391 overweight and obese women with a body mass index (BMI) ranging from 25 to 40 kg/m^2^ and aged 18–48 years, recruited from the community health center of the Tehran University of Medical Sciences (TUMS) in 2018. We used the sample size formula *N* =([(Z _1−α+_Z_1−β_)$\times \sqrt{}$1-r^2^]/r)^2^+2), β = 95%, and α = 0.05, *r* = 0.25. Participants were excluded from the study if they reported a total daily energy intake outside of 800–4,200 kcal (17,556–3,344 kJ) (23) or if they reported a history of diseases such as CVD, diabetes, cancer, kidney disease, thyroid disease, menopause, pregnancy, and breastfeeding. In addition, individuals on lipid-lowering agents, individuals on blood glucose-lowering agents, and those who consumed alcohol or smoked were excluded from the study. Furthermore, the food frequency questionnaire (FFQ) did not include individuals who did not respond to more than 70 questions and had significant fluctuations in their weight over the past year. After learning about the study's objectives, all the participants signed an informed consent form. The Human Ethics Committee of Tehran University of Medical Sciences approved the study protocol (Ethics number: IR.TUMS.VCR.REC.1398.142, Date of reference number: 5 April 2019).

### Dietary assessment and NOVA calculation

To evaluate the food consumption of participants during the previous year, we used a validated semi-quantitative FFQ, whose validity and reliability have already been authorized (24, 25). Trained dietitians were responsible for applying the FFQ. In total, one hundred forty-seven food items were included in this FFQ with a standard serving size, and participants assessed their consumption frequency according to four categories: daily, weekly, monthly, and infrequent. Using home measures, the portion sizes of the consumed foods were converted to grams (23). Nutrient and energy intakes were calculated using NUTRITIONIST IV software (version 7.0; N-Squared Computing, Salem, OR). The following food and beverage items are classified as UPFs in the NOVA food group classification, which is the subject of this research, and are grouped into the FFQ into seven food groups (daily intake was calculated as grams): (1) Non-dairy beverages (coffee, cola, nectar, and industrial sweet drink), (2) dairy beverages (ice cream, pasteurized and non-pasteurized, chocolate milk, and cocoa milk), (3) cakes and cookies (cookies, biscuits, pastries (creamy and non-creamy), cake, pancake, industrial bread, toasted bread, noodles, and pasta), (4) fast food and processed meat (burger, sausage, pizza, and bologna), (5) salty snacks (chips, crisps, crackers, and cheese puff), (6) oil and sauce (mayonnaise, margarine, and ketchup), (7) sweets (Gaz, Sohan, Noghl, sesame halva, chocolate, candies, rock candies, jam, and sweets) (26). All the NOVA components were adjusted for energy intake.

### Anthropometry and body composition

Participants were advised to fast for 12 h the night before the assessment and avoid unusual physical activity for 72 h before the anthropometrics and body composition assessments. A digital stadiometer (Seca) was used to measure height (m) with a precision of 0.5 cm. The waist circumference (WC) (cm) and hip circumference (HC) (cm) with an accuracy of 0.5 cm were measured within the largest and the littlest circumference separately. The waist-to-hip ratio (WHR) was computed as WC (cm)/HC (cm).

A multi-frequency bioelectric impedance analyzer (BIA) (Inbody Co., Seoul, Korea) scanner evaluated body composition. This electrical impedance analyzer measures the resistance of body tissues to the passage of an electrical signal given through the feet and hands. The body composition analyzer was used to assess the individuals' weight, BMI, fat mass (FM), fat-free mass (FFM), body fat percentage (%), and the others, according to a predetermined methodology. The participants were instructed to urinate before measuring their body composition according to the fabricant recommendations.

### Biochemical assessment

The blood samples were obtained between 8:00 and 10:00 a.m. at the Nutrition and Biochemistry lab of the School of Nutritional Sciences and Dietetics, TUMS, after an overnight fast and deposited in tubes containing 0.1 percent ethylenediaminetetraacetic acid (EDTA). The serum was centrifuged, aliquoted, and stored at −70°C. The glucose oxidase phenol 4-aminoantipyrine peroxidase (GOD/PAP) technique determined fasting blood glucose levels (FBG). To evaluate blood triglyceride (TG) levels, enzyme colorimetric assays with GPO–PAP were utilized. Total cholesterol was assessed using phenol 4-aminoantipyrine peroxidase (CHOD–PAP), low-density lipoprotein (LDL), and high-density lipoprotein (HDL) were measured using the direct approach and immunoinhibition. The serum high-sensitivity C-reactive protein (hs-CRP) was measured using an immunoturbidimetric method. The Enzyme-Linked Immunosorbent Assays (ELISA) technique was used to evaluate the levels of IL-1β and PA-I (Human PAI-1^*^96 T ELIZA kit Crystal Company). The serum insulin concentrations were determined using the enzyme-linked immunosorbent assay (ELISA kit). The ELISA kit was also used to quantify serum MCP-1 levels (Zell Bio GmbH, Germany, assay range:5 ng/L−1,500 ng/L, sensitivity:2.4 ng/L, CV10 percent inter-assay variability). All of the kits were given by Pars Azmoon (Pars Azmoon Inc. Tehran, Iran). Insulin resistance was assessed using a homeostasis model (HOMA–IR). The index was computed using the algorithm (plasma glucose mmol/ l / × fasting plasma insulin mIU/ l)/22.5 (27). The quantitative insulin sensitivity check index (QUICKI) was also used to evaluate insulin resistance through the formula 1/[log (fasting insulin) + log (fasting glucose)] (27). From biochemical parameters, FBG, TG, HDL, LDL, hs-CRP, and IL_1β variables are considered as CVD risk factors in this study.

The atherogenic index of plasma (AIP) was calculated using the logarithmic of (TG/HDL-C). TC/HDL, LDL/HDL, and (TC-HDL) /LDL were used to determine castelli's risk index 1 (CRI- I), castelli's risk index 2 (CRI- II), and atherogenic coefficient (AC), respectively. The following formula was used to compute CHOLIndex: CHOLIndex = LDL-C – HDL-C (TG <400) = LDL-C – HDL-C + 1/5 TG (TG >400). (28). Ln (FBG (mg/dl) ^*^ TG (mg/dl)/2) was used to determine triglyceride–glucose index (TyG index) (29). The terms triglyceride glucose-waist circumference (TyG–WC) and triglyceride glucose–body mass index (TyG–BMI) were obtained through the formulas: [Ln (FBG (mg/dl) ^*^ TG (mg/dl)/2)] ^*^ WC and [Ln (FBG (mg/dl) ^*^ TG (mg/dl)/2)] ^*^ BMI), respectively (30).

### Blood pressure assessment

Blood pressure was measured using an automated sphygmomanometer according to standard procedures (OMRON, Germany).

### Other collected data

General information about the participants, such as their age, job status (employed, unemployed), education level (illiterate, under diploma, diploma, and bachelor and higher) (what are the categories? Detail the methodology here, as well as the other variables), marital status (single and married), economic status (low, middle, and high class), standard questionnaires, were collected. The physical activity status was obtained using the validated International Physical Activity Questionnaire (IPAQ). Afterward, metabolic equation hours per day (MET-min/week) were calculated for each subject. After that, each subject's metabolic equation hours per day score (MET-min/week) was calculated. Trained professionals were responsible for applying the questionnaires (31, 32).

### Statistical analyses

The Kolmogorov–Smirnov test was used to check the quantitative variable's normality (*P* > 0.05). Categorical data were reported as absolute and relative frequencies, and quantitative data were reported as means and standard deviation (SD). According to the NOVA score, the participants were categorized into tertiles of UPF consumption in grams. To compare the mean difference of quantitative and frequency of categorical variables across UPF tertiles, a one-way analysis of variance (ANOVA) and Pearson chi-square (χ^2^) tests were performed, respectively. Analysis of covariance (ANCOVA) adjusted for potential confounders (age, BMI, energy intake, and physical activity) and considering BMI as a collinear variable for anthropometrics and body composition variables were performed. The Bonferroni *post-hoc* test was used to detect the statistically significant difference among UPF tertiles. Linear regression was performed to evaluate the association of the UPF consumption (independent variable) with cardiometabolic risk factors (dependent variable). Model 1 was adjusted for age, BMI, physical activity, total energy intake, supplements intake, and job status. Model 2 was further adjusted for legumes and vegetables. This analysis was presented as the β-value and a confidence interval of 95% (CI). SPSS v.26 software (SPSS Inc., IL, USA) was used for statistical analysis. The significance level was set at *p* < 0.05.

## Results

A total of 391 participants were included in the present study. Women had a mean (SD) age of 36.67 (9.10) years and a mean BMI of 31.26 (4.29) kg/m^2^. The majority of women were employed (97%), 47% were highly educated (bachelor's degree and higher), and 45.5% had a middle income. The mean of UPF intake in our sample was 442.47 (127.91) g or 96.8 %.

The general characteristics of participants among UPF tertiles are presented in Table 1. The average UPF consumption in tertile 1 was <383,681 g, in tertile 2 was from 383,681 g to 467,713 g, and in tertile 3 was >467,713 g. The mean of age (*P* = 0.003) was statistically different between UPF tertiles in the crude model and after controlling for confounding variables. The mean height (*P* = 0.047) was statistically different between UPF tertiles in the crude model. According to the Bonferroni's *post-hoc* test, the significant mean difference in age was between T2 and T3, and the mean difference was higher in T2 than in T3. In the categorical variables, the supplementation intake (*P* = 0.057) and job status (*P* = 0.073) were marginally significant between UPF tertiles after controlling for cofounders. There was no significant difference for other variables (Table 1).

**Table 1:** General characteristics among tertiles of NOVA score in obese and overweight women (*n* = 391).

**Quantitative variables**	**NOVA tertiles**			***P*-value**	*P*-value*
	**T1**	**T2**	**T3**		
	**<383.681**	**383.681–467.713**	**>467.713**		
	***N* = 131**	***N* = 130**	***N* = 130**		
	**Mean** ±**SD**				
**Age (year)** ^a^	36.480 ± 9.138	38.759 ± 8.77	34.860 ± 9.352	**0.003**	**0.004**
**PA (MET-min -week)**	1,465.171 ± 231.881	834.995 ± 235.775	1,353.665 ± 254.709	0.098	0.154
**Weight (kg)**	81.958 ± 12.382	79.884 ± 10.975	81.669 ± 13.320	0.337	0.365
**Height (cm)**	161.574 ± 5.888	160.115 ± 5.881	161.763 ± 5.796	**0.047**	0.869
**BMI (kg/m** ^**2**^ **)**	31.141 ± 0.440	30.847 ± 0.449	30.459 ± 0.483	0.946	0.576
**WC (cm)**	113.163 ± 8.516	113.638 ± 7.477	116.295 ± 13.637	0.247	0.592
**BMC (Kg)**	2.676 ± 0.376	2.622 ± 0.342	2.661 ± 0.330	0.445	0.643
**SMM (Kg)**	25.954 ± 3.281	25.347 ± 3.300	25.333 ± 3.4205	0.247	0.311
**SLM (Kg)**	44.083 ± 5.759	43.585 ± 5.126	43.587 ± 5.317	0.693	0.454
**Categorical variables**					
**Supplementation intake** ***n*** **(%)**^**b**^				0.311	**0.057**
Yes %	58 (36.7)	47 (29.7)	53 (33.5)		
No %	51 (29.0)	61 (34.7)	64 (36.4)		
**Income status** ***n*** **(%)**				0.582	0.185
Low class	33 (37.5)	31 (35.2)	24 (27.3)		
Middle class	60 (33.0)	61 (33.5)	61 (33.5)		
High class	35 (32.7)	31 (29.0)	41 (38.3)		
**Marital status** ***n*** **(%)**				0.275	0.880
Single	35 (32.1)	31 (28.4)	43 (39.4)		
Married	92 (33.6)	96 (35)	86 (31.4)		
**Job status** ***n*** **(%)**				0.137	**0.073**
Unemployed	2 (100)	0 (0)	0 (0)		
Employed	128 (33.2)	129 (33.5)	128 (33.2)		
**Educational status** ***n*** **(%)**				0.753	0.744
Illiterate	1 (25)	1 (25)	2 (50)		
Under diploma	12 (26.1)	17 (37)	17 (37)		
Diploma	46 (30.9)	54 (36.2)	49 (32.9)		
Bachelor and higher	68 (37)	55 (29.9)	61 (33.2)		

PA, physical activity; BMI, body mass index; WC, waist circumference; BMC, bone mineral content; SMM, skeletal muscle mass; SLM, soft lean mass.

Values are represented as means and SD and number (%) for categorical variables.

ANCOVA (P-value^*^) was performed to adjust potential confounding factors; age, energy intake, PA, BMI. BMI consider as the collinear variable for body composition, and anthropometric measurements.

p <0.05 were considered as significant.

a significant difference was seen between T3 and T2.

b significant difference was seen between T2 and T3.

A p < 0.05 were considered as significant and p-values of 0.05, 0.06, and 0.07 were considered as marginally significant.

### Dietary intakes among the UPF tertiles

Dietary intakes of all the participants among tertiles of UPF consumption are presented in Table 2. The mean of non-dairy beverages (*P* = 0.001), dairy beverages (*P* = 0.001), cookies (cakes) (*P* = 0.001), potato chips (salty) (*P* = 0.001), processed meat (fast food) (*P* = 0.001), oil (sauce group) (*P* = 0.005), sweet (*P* = 0.001) were statistically different among UPF tertiles, with it being higher in the third tertile. With increasing UPF consumption, non-dairy beverages, cookies (cakes), dairy beverages, potato chips (salty), processed meat (fast food), oil, sauce, and sweet have increased in the crude and adjusted model.

**Table 2:** Dietary intakes among tertiles of the NOVA score in obese and overweight women (*n* = 391).

	**Total**	**UPF consumption tertiles**			***P*-value**	*P*-value*
		**T1 (*n* = 131)** **<383.681**	**T2 (*n* = 130) 383.681-467.713**	**T3 (*n* = 130)** **>467.713**		
**NOVA score components**	
Nondairy beverages (g/d)	177.351 ± 93.223	124.069 ± 25.540	157.152 ± 27.648	251.242 ± 126.711	**0.001**	**0.001**
Cookies-cakes (g/d)	98.913 ± 44.205	75.570 ± 25.626	97.288 ± 28.007	124.061 ± 57.167	**0.001**	**0.001**
Dairy beverages (g/d)	47.833 ± 27.952	37.472 ± 18.4894	46.629 ± 22.117	59.479 ± 35.795	**0.001**	**0.001**
Potato chips- salty	22.106 ± 13.893	17.354 ± 9.094	22.652 ± 10.166	26.348 ± 18.853	**0.001**	**0.001**
Processed meat- fast food (g/d)	41.138 ± 25.424	28.402 ± 12.600	40.230 ± 14.202	54.881 ± 35.167	**0.001**	**0.001**
Oil_ Sause (g/d)	18.269 ± 8.727	16.764 ± 8.5494	17.861 ± 7.4184	20.194 ± 9.766	**0.005**	**0.005**
Sweet (g/d)	36.861 ± 24.0635	30.679 ± 15.1176	36.916 ± 17.1858	43.037 ± 33.8778	**0.001**	**0.001**
**Food groups**
Refined grains (g/d)	432.348 ± 220.133	474.142 ± 191.103	380.801 ± 207.529	444.129 ± 253.5120	**0.008**	0.969
Whole grains (g/d)	7.586 ± 10.410	9.144 ± 11.2396	6.769 ± 9.0196	6.746 ± 10.831	0.177	0.361
Fruits (g/d)	528.904 ± 338.1681	605.778 ± 317.153	466.287 ± 317.377	513.252 ± 370.044	**0.011**	0.340
Vegetables (g/d)	433.577 ± 263.259	526.618 ± 264.203	382.927 ± 226.814	385.498 ± 275.073	**0.001**	**0.003**
Nuts (g/d)	14.370 ± 16.1868	17.821 ± 17.786	11.449 ± 14.354	13.795 ± 15.697	**0.018**	0.518
Legumes (g/d)	52.691 ± 41.2788	63.432 ± 49.5718	45.834 ± 35.7690	48.313 ± 34.0807	**0.005**	**0.045**
Dairy (g/d)	387.451 ± 246.357	438.192 ± 267.952	330.196 ± 224.147	394.927 ± 233.413	**0.007**	0.769
Eggs (g/d)	21.687 ± 14.174	22.105 ± 12.3656	21.235 ± 12.394	21.732 ± 17.7520	0.909	0.569
Fish and seafood (g/d)	11.408 ± 12.1569	12.086 ± 11.932	10.743 ± 11.2257	11.399 ± 13.4774	0.735	0.990
Meats (g/d)	64.571 ± 50.1758	67.371 ± 40.9762	54.081 ± 41.6793	73.518 ± 65.0100	**0.022**	0.250
Red meat (g/d)	21.479 ± 18.5197	24.003 ± 20.368	17.760 ± 15.8117	22.894 ± 18.722	**0.038**	0.947
**Macronutrients and energy**
Energy intake (kcal/d)	2633.280 ± 809.432	2916.675 ± 654.474	2267.608 ± 712.433	2713.37 ± 904.867	**0.001**	-
**Micronutrients**
SFA (mg/d)	28.409 ± 11.545	30.861 ± 11.417	24.761 ± 10.291	29.587 ± 12.033	**0.001**	0.628
MUFA (mg/d)	32.008 ± 12.917	35.155 ± 13.593	27.591 ± 10.563	33.253 ± 13.241	**0.001**	0.817
PUFA (mg/d)	20.082 ± 9.568	22.589 ± 10.515	17.403 ± 8.316	20.235 ± 9.087	**0.001**	0.717
Trans fat (g/d)	0.0007 ± 0.002	0.001 ± 0.003	0.0006 ± 0.001	0.0005 ± 0.001	0.097	0.120
Total fiber (g/d)	47.344 ± 21.360	57.263 ± 21.377	40.359 ± 19.203	44.333 ± 19.795	0.078	**0.001**

pro, protein; Cho, carbohydrate; SAFA, saturated fatty acid; MUFA, monounsaturated fatty acid; PUFA, polyunsaturated fatty acid.

Values are represented as means (SD).

ANCOVA (P-value^*^) was performed to adjust potential confounding factors (energy intake).

A P-value under 0.05 is considered significant.

### CVD risk factors among the UPF tertiles

The association of CVD risk factors among UPF consumption tertiles is shown in Table 3. UPF consumption was associated with the HOMA–IR index (*P* = 0.024), hs-CRP (*P* = 0.001), and TYG–WC (*P* = 0.026). On the contrary, it was marginally associated with the markers TGF (*P* = 0.077), AC (*P* = 0.072), CRI-1 (*P* = 0.062), and NC (*P* = 0.068).

**Table 3:** CVD risk factors consist of anthropometric measurements and body composition, biochemical variables, and inflammatory factors among tertiles of NOVA score in obese and overweight women (*n* = 391).

**Variables**		**UPF consumption tertiles**			***P*-value**
		**T1 <383.681**	**T2 383.681– 467.713**	**T3 >467.713**	
**Body Composition**					
FFM (Kg)	Crude	47.019 ± 5.938	46.217 ± 5.444	46.263 ± 5.616	0.440
	Model 1	46.402 ± 0.982	47.858 ± 1.037	46.017 ± 1.332	0.513
	Model 2	46.286 ± 1.014	47.917 ± 1.058	46.121 ± 1.347	0.499
FFMI	Crude	18.977 ± 1.618	17.977 ± 1.443	17.672 ± 11.450	0.266
	Model 1	17.801 ± 0.246	18.153 ± 0.260	17.838 ± 0.334	0.625
	Model 2	17.729 ± 0.252	18.202 ± 0.263	17.882 ± 0.33	0.479
FMI	Crude	13.422 ± 3.163	13.318 ± 3.235	13.610 ± 3.799	0.784
	Model 1	12.214 ± 0.590	12.903 ± 0.623	12.217 ± 0.800	0.716
	Model 2	12.168 ± 0.612	12.929 ± 0.638	12.255 ± 0.813	0.700
BF (%)	Crude	42.238 ± 5.016	41.890 ± 5.255	42.550 ± 6.196	0.629
	Model 1	40.208 ± 1.026	41.033 ± 1.084	39.571 ± 1.392	0.725
	Model 2	40.174 ± 1.066	41.058 ± 1.112	39.588 ± 1.416	0.726
BFM (Kg)	Crude	34.936 ± 8.395	33.830 ± 7.801	35.421 ± 9.887	0.325
	Model 1	31.494 ± 1.421	33.926 ± 1.501	31.150 ± 1.927	0.450
	Model 2	31.387 ± 1.471	33.978 ± 1.535	31.251 ± 1.955	0.450
TF (kg)	Crude	16.965 ± 3.489	16.5070 ± 3.411	17.103 ± 4.086	0.393
	Model 1	15.609 ± 0.624	16.608 ± 0.660	15.450 ± 0.847	0.493
	Model 2	15.571 ± 0.648	16.630 ± 0.676	15.479 ± 0.861	0.492
TF (%)	Crude	320.916 ± 65.872	317.959 ± 68.968	322.384 ± 75.379	0.875
	Model 1	298.070 ± 12.616	312.018 ± 13.326	297.691 ± 17.107	0.736
	Model 2	297.023 ± 13.092	312.746 ± 13.660	298.300 ± 17.394	0.712
**Anthropometric measurements**					
WC (cm)	Crude	97.281 ± 16.058	97.138 ± 12.693	97.951 ± 17.058	0.933
	Model 1	92.021 ± 3.239	98.703 ± 3.421	89.857 ± 4.391	0.259
	Model 2	91.161 ± 3.329	99.287 ± 3.473	90.382 ± 4.423	0.207
WHR	Crude	0.939 ± 0.054	1.637 ± 8.018	0.936 ± 0.051	0.372
	Model 1	0.931 ± 0.009	0.940 ± 0.010	0.911 ± 0.013	0.236
	Model 2	0.931 ± 0.010	0.939 ± 0.010	0.911 ± 0.013	0.233
VFA (CM^2^)	Crude	168.858 ± 36.720	176.087 ± 150.293	168.733 ± 42.799	0.764
	Model 1	154.422 ± 6.889	161.912 ± 7.276	147.917 ± 9.341	0.526
	Model 2	154.298 ± 7.155	161.986 ± 7.465	148.012 ± 9.506	0.536
VFL	Crude	17.122 ± 12.037	15.612 ± 3.307	17.514 ± 17.260	0.423
	Model 1	14.815 ± 0.605	15.404 ± 0.639	14.262 ± 0.820	0.576
	Model 2	14.826 ± 0.628	15.397 ± 0.656	14.252 ± 0.835	0.589
NC ^a^ (cm)	Crude^a^	38.338 ± 12.042	36.958 ± 2.702	37.430 ± 3.942	0.537
	Model 1^b^	36.130 ± 0.421	37.791 ± 0.445	36.420 ± 0.571	**0.036**
	Model 2^a^	36.233 ± 0.433	37.723 ± 0.452	36.354 ± 0.575	**0.068**
**Biochemical variables**					
SBP (mmHg)	Crude	113.000 ± 15.0006	112.227 ± 12.386	108.333 ± 17.190	0.083
	Model 1	113.074 ± 1.661	111.479 ± 1.691	108.975 ± 1.734	0.231
	Model 2	113.374 ± 1.4497	110.824 ± 1.504	119.552 ± 1.164	0.222
DBP (mmHg)	Crude	77.969 ± 9.586	77.930 ± 9.454	76.547 ± 12.418	0.590
	Model 1	77.745 ± 1.194	77.736 ± 1.216	77.931 ± 1.247	0.992
	Model 2	78.237 ± 1.064	76.992 ± 1.069	78.117 ± 1.172	0.677
HOMA-IR	Crude	3.240 ± 1.346	3.585 ± 1.388	3.142 ± 1.007	**0.073**
	Model 1	2.941 ± 0.190	3.770 ± 0.189	3.143 ± 0.204	0.011
	Model 2^a^	3.031 ± 0.196	3.738 ± 0.189	3.078 ± 0.207	**0.024**
Insulin (mIU/ ml)	Crude	1.205 ± 0.245	1.240 ± 0.234	1.194 ± 0.197	0.415
	Model 1	1.229 ± 0.035	1.235 ± 0.034	1.190 ± 0.037	0.636
	Model 2^a^	1.227 ± 0.036	1.237 ± 0.035	1.189 ± 0.038	0.629
FBG (mg/dL)	Crude	87.569 ± 9.927	88.912 ± 10.343	85.536 ± 7.919	0.089
	Model 1	84.834 ± 1.382	88.262 ± 1.372	84.393 ± 1.482	0.126
	Model 2	85.057 ± 1.439	88.199 ± 1.388	84.209 ± 1.516	0.136
TC (mg/dL)	Crude	182.383 ± 37.483	189.395 ± 33.851	183.000 ± 37.733	0.371
	Model 1	177.717 ± 4.406	179.295 ± 4.417	184.293 ± 466	0.569
	Model 2	177.026 ± 4.583	179.669 ± 4.472	184.648 ± 4.741	0.514
TG (mg/dL)	Crude	118.267 ± 55.944	120.022 ± 59.970	116.144 ± 64.776	0.922
	Model 1	115.533 ± 8.472	126.502 ± 8.494	118.257 ± 8.966	0.666
	Model 2	114.907 ± 4.583	179.669 ± 8.609	118.752 ± 9.126	0.653
HDL (mg/dL)	Crude	47.267 ± 10.965	47.340 ± 11.662	45.536 ± 9.567	0.518
	Model 1	48.039 ± 1.328	45.996 ± 1.331	47.130 ± 1.405	0.584
	Model 2	48.149 ± 1.383	45.941 ± 1.350	47.068 ± 1.431	0.561
LDL (mg/dL)	Crude	95.244 ± 23.856	97.109 ± 24.795	92.029 ± 23.850	0.420
	Model 1	98.986 ± 3.071	98.070 ± 3.078	99.803 ± 3.249	0.931
	Model 2	98.176 ± 3.186	98.486 ± 3.109	100.248 ± 3.296	0.890
GOT (mg/dL)	Crude	17.720 ± 7.441	18.604 ± 8.179	16.927 ± 5.976	0.358
	Model 1	18.502 ± 1.037	18.579 ± 1.021	16.154 ± 1.104	0.202
	Model 2	18.365 ± 1.064	18.675 ± 1.022	16.194 ± 1.112	0.221
GPT (mg/dL)	Crude	19.209 ± 14.249	20.373 ± 13.793	17.478 ± 9.842	0.378
	Model 1	21.013 ± 1.886	21.034 ± 1.857	16.240 ± 2.008	0.144
	Model 2	21.125 ± 1.945	21.047 ± 1.868	16.093 ± 2.033	0.131
AIP	Crude	0.366 ± 0.236	0.362 ± 0.240	0.361 ± 0.272	0.990
	Model 1	0.343 ± 0.034	0.403 ± 0.034	0.353 ± 0.036	0.449
	Model 2	0.339 ± 0.035	0.404 ± 0.034	0.356 ± 0.036	0.434
CRI-I	Crude	4.029 ± 1.206	4.194 ± 1.209	4.294 ± 2.100	0.542
	Model 1	3.778 ± 0.118	3.998 ± 0.118	4.025 ± 0.125	0.292
	Model 2	3.755 ± 0.122	4.010 ± 0.119	4.038 ± 0.127	**0.062**
CRI-II	Crude	2.075 ± 0.531	2.132 ± 0.642	2.075 ± 0.580	0.765
	Model 1	2.114 ± 0.080	2.191 ± 0.080	2.187 ± 0.085	0.760
	Model 2	2.091 ± 0.083	2.202 ± 0.081	2.200 ± 0.086	0.593
AC	Crude	3.029 ± 1.206	3.194 ± 1.209	3.294 ± 2.100	0.542
	Model 1	2.778 ± 0.118	2.998 ± 0.118	3.025 ± 0.125	0.292
	Model 2	2.755 ± 0.122	3.010 ± 0.119	3.038 ± 0.127	**0.072**
CHOLIndex	Crude	47.976 ± 21.460	49.769 ± 23.040	46.492 ± 23.306	0.657
	Model 1	50.947 ± 3.040	52.074 ± 3.048	52.674 ± 3.218	0.925
	Model 2	50.027 ± 3.151	52.545 ± 3.075	53.180 ± 3.260	0.775
TyG index	Crude	8.446 ± 0.478	8.466 ± 0.486	8.377 ± 0.494	0.516
	Model 1	8.404 ± 0.068	8.517 ± 0.068	8.402 ± 0.072	0.434
	Model 2	8403 ± 0.070	8.517 ± 0.069	8.404 ± 0.73	0.449
TyG-BMI	Crude	261.876 ± 40.206	261.827 ± 41.224	255.425 ± 46.928	0.571
	Model 1	253.760 ± 7.272	246.303 ± 7.625	254 ± 337 ± 8.691	0.586
	Model 2	252.295 ± 7.491	265.151 ± 7.716	255.342 ± 8.813	0.510
TyG-WC	Crude	810.989 ± 177.111	812.087 ± 135.178	791.246 ± 132.359	0.76
	Model 1^b^	718.567 ± 26.978	838.143 ± 28.287	754.874 ± 32.243	**0.057**
	Model 2^b^	741.185 ± 27.740	842.278 ± 28.573	760.139 ± 32.637	**0.026**
**Inflammatory biomarkers**					
PAL-1 (mg/dl)	Crude	20.265 ± 39.585	14.200 ± 24.731	13.319 ± 20.377	0.405
	Model 1	31.411 ± 12.591	23.991 ± 15.100	5.875 ± 12.168	0.429
	Model 2	47.091 ± 17.465	20.579 ± 18.555	9.139 ± 12.762	0.255
MCP1 (mg/dl)	Crude	57.514 ± 94.785	54.332 ± 109.983	36.967 ± 54.657	0.389
	Model 1	83.110 ± 28.588	52.148 ± 34.285	25.798 ± 27.629	0.301
	Model 2	117.021 ± 40.136	57.515 ± 42.640	25.996 ± 29.328	0.224
TGF (ng/ml)	Crude	74.436 ± 39.046	80.671 ± 61.1695	80.775 ± 41.250	0.733
	Model 1	55.998 ± 10.530	78.067 ± 12.628	88.626 ± 10.177	0.295
	Model 2^c^	49.350 ± 15.871	78.440 ± 16.862	88.086 ± 11.597	**0.077**
IL_1β (ng/ml)	Crude	2.585 ± 0.895	2.745 ± 1.022	2.843 ± 0.927	0.647
	Model 1	2.625 ± 0.274	3.010 ± 0.328	2.715 ± 0.264	0.538
	Model 2	2.307 ± 0.418	3.052 ± 0.445	2.708 ± 0.306	0.580
hs_CRP (mg/l)	Crude	4.300 ± 4.624	4.219 ± 4.641	4.480 ± 4.773	0.942
	Model 1^b^	3.905 ± 0.727	1.385 ± 0.871	6.109 ± 0.702	**0.001**
	Model 2 ^b^	3.972 ± 1.006	0.566 ± 1.069	6.390 ± 0.735	**0.001**

AC, atherogenic coefficient; BFM, body fat mass; BF, body fat; FFM, fat-free mass; FMI, fat mass index; FFMI, fat-free mass index; WC, waist circumference; WHR, Waist-to-Hip Ratio; NC, Neck circumference; IL-1β, interleukin-1 beta; MCP-1, monocyte chemoattractant protein-1; CRI, Cardiac risk index; SBP, Systolic Blood Pressure; DBP, Diastolic Blood Pressure; FBS, Fasting Blood Sugar; TG, Triglyceride; HDL, High-density lipoprotein; LDL, Low-density lipoprotein; GPT, Glutamic-pyruvic transaminase; GOT, Glutamic-oxaloacetic transaminase; PAI-1, plasminogen activator inhibitor- 1, TF, Trunk Fat, VFA, Visceral fat area, VFL, Visceral fat level, SD Standard deviation; hs-CRP, high sensitive- C reactive protein; TC, Total cholesterol; AIP, Atherogenic index of plasma; TyG, Triglyceride-glucose; TGF, Transforming growth factor.

Quantitative variables were shown by means ± SD and categorical variables were shown by number (%).

P-values resulted from one-way ANOVA analysis and thechi-squared test. A p-value < 0.05 was considered significant and p-values equal to 0.05, 0.06, and 0.07 were considered marginally significant.

*P-values resulted from ANCOVA analysis and were adjusted.

Model 1: Adjusted for age, BMI, physical activity, total energy intake, supplements intake, and job status (BMI consider as a collinear variable).

Model 2: Additionally controlled for the effect of vegetables and legumes.

The Bonferroni post-hoc test was used to investigate differences between tertiles, significant difference between two means with p < 0.05. 0.05, and 0.06 considered as marginally significant.

a Significant difference was seen between T1 and T2.

b Significant difference was seen between T2 and T3.

c Significant difference was seen between T1 and T3.

### Association between UPF consumption and CVD risk factors, anthropometric measurements, body composition, biochemical variables, and inflammatory factors

Association between UPF consumption and CVD risk factors, anthropometric measurements, body composition, biochemical variables, and inflammatory factors in crude and adjusted models present with β-value and a 95% CI is shown in Table 4. In the model 1, there was a significant association between UPF consumption and TGF (β: 0.101, 95% CI: 0.023, 0.180, *p* = 0.012). Also, there was a significant association between UPF consumption and AC (β: 0.011, 95%CI: 0.001, 0.032, *p* = 0.034), VLF (β: 0.006, 95% CI: −0.017, 0.029, *p* = 0.076), and ISQIUKI (β: −3.775, 95% CI: 0.001, 0.001, *P* = 0.042). With increasing one gram of UPF intake, AC increases to 0.011, VFL increases by 0.006, and QUICKI is significantly reduced by −3.775 mg/lit. The other variables in Table 3 had no significant association.

**Table 4:** Association between NOVA score and CVD risk factors, anthropometric measurements, body composition, biochemical variables, and inflammatory factors in obese and overweight women (*n* = 391).

**Variables**		**NOVA score**		***P*-value**	*P*-value*
		**β (SE)**	**CI (95%)**	
**Body composition**					
FFM (Kg)	Crude	−0.001 (0.002)	−0.005, 0.003	0.682	-
	Model 1	−0.004 (0.004)	−0.011, 0.003	-	0.278
	Model 2	−0.004 (0.004)	0.001, 0.001	-	**0.056**
FFMI	Crude	−0.004 (0.003)	−0.009, 0.001	0.115	-
	Model 1	−0.001 (0.001)	−0.003, 0.001	-	0.201
	Model 2	−0.001 (0.001)	−0.003, 0.001	-	0.376
FMI	Crude	−0.004 (0.001)	−0.014, 0.006	0.427	-
	Model 1	0.001 (0.002)	−0.005, 0.004	-	0.878
	Model 2	−5.286 (0.002)	−0.004, 0.004	-	0.981
BF (%)	Crude	0.003 (0.002)	−0.001, 0.007	0.148	-
	Model 1	0.001 (0.004)	−0.006, 0.008	-	0.768
	Model 2	0.001 (0.004)	−0.006, 0.008	-	0.768
BFM (Kg)	Crude	0.006 (0.003)	−0.001, 0.013	0.084	-
	Model 1	−0.002 (0.006)	−0.013, 0.009	-	0.719
	Model 2	−0.002 (0.006)	−0.014, 0.009	0.658	
TF (kg)	Crude	0.002 (0.001)	0.001, 0.005	0.097	-
	Model 1	−0.001 (0.002)	−0.005, 0.004	-	0.812
	Model 2	−0.001 (0.002)	−0.006, 0.004	-	0.709
TF (%)	Crude	0.035 (0.028)	−0.020, 0.089	0.213	-
	Model 1	0.001 (0.044)	−0.086, 0.088	-	0.988
	Model 2	−0.005 (0.045)	−0.094, 0.083	-	0.904
VFA (CM^2^)	Crude	0.028 (0.037)	−0.044, 0.100	0.442	-
	Model 1	−0.005 (0.082)	−0.168, 0.157	-	0.951
	Model 2	−0.007 (0.085)	−0.174, 0.159	-	0.930
VFL	Crude	0.002 (0.005)	−0.007, 0.012	0.647	-
	Model 1	0.005 (0.011)	−0.017, 0.028	-	0.651
	Model 2	0.006 (0.012)	−0.017, 0.029	-	**0.076**
**Biochemical variables**					
Insulin (mIU/ml)	Crude	−6.160 (0.001)	0.001, 0.001	0.617	-
	Model 1	0.001 (0.001)	−0.001, 0.000	-	0.230
	Model 2	0.001 (0.001)	0.001, 0.001	-	0.273
HOMA_IR	Crude	0.001 (0.001)	−0.0002, 0.001	0.671	-
	Model 1	−2.096 (0.001)	−0.002, 0.002	-	0.981
	Model 2	0.001 (0.001)	0.001, 0.033	-	**0.055**
QUICKI (mg/lit)	Crude	−1.731 (0.001)	0.001, 0.001	0.205	-
	Model 1	−4.306 (0.001)	−0.001, 0.001	-	0.720
	Model 2	−3.775 (0.001)	0.001, 0.001	-	**0.042**
hs-CRP (mg/l)	Crude	0.001 (0.003)	−0.005, 0.005	0.962	-
	Model 1	0.001 (0.003)	−0.006, 0.007	-	0.875
	Model 2	0.001 (0.003)	−0.006, 0.007	-	0.916
FBG (mg/dL)	Crude	−0.004 (0.005)	−0.014, 0.006	0.427	-
	Model 1	−0.004 (0.006)	−0.017, 0.009	-	0.506
	Model 2	−0.006 (0.007)	−0.020, 0.007	-	0.335
SBP (mmHg)	Crude	−0.012 (0.007)	−0.027, 0.002	0.102	-
	Model 1	−0.015 (0.008)	−0.032, 0.002	-	0.032
	Model 2	0.017 (0.008)	−0.001, 0.020	-	0.148
DBP (mmHg)	Crude	−0.008 (0.005)	−0.018, 0.002	0.134	-
	Model 1	−0.004 (0.086)	−0.016, 0.008	-	0.503
	Model 2	−0.007 (0.006)	−0.019, 0.005	-	0.236
TC (mg/dL)	Crude	0.003 (0.019)	−0.036, 0.041	0.893	-
	Model 1	0.012 (0.022)	−0.032, 0.055	-	0.598
	Model 2	0.020 (0.022)	−0.024, 0.064	-	**0.073**
TG (mg/dL)	Crude	0.003 (0.032)	−0.060, 0.067	0.916	-
	Model 1	0.031 (0.042)	−0.052, 0.115	-	0.456
	Model 2	0.041 (0.043)	−0.044, 0.126	-	0.344
HDL (mg/dL)	Crude	−0.005 (0.006)	−0.016, 0.006	0.391	-
	Model 1	−0.004 (0.007)	−0.017, 0.010	-	0.588
	Model 2	−0.002 (0.007)	−0.016, 0.011	-	0.720
LDL (mg/dL)	Crude	−0.010 (0.013)	−0.036, 0.015	0.433	-
	Model 1	−6.043 (0.015)	−0.030, 0.030	-	0.997
	Model 2	0.007 (0.016)	−0.024, 0.038	-	0.662
GOT (mg/dL)	Crude	−0.004 (0.004)	−0.012, 0.0003	0.274	-
	Model 1	−0.007 (0.005)	−0.017, 0.003	-	0.167
	Model 2	−0.006 (0.005)	−0.017, 0.004	-	0.245
GPT (mg/dL)	Crude	−0.006 (0.007)	−0.020, 0.008	0.391	-
	Model 1	−0.016 (0.009)	−0.034, 0.003	-	0.097
	Model 2	−0.015 (0.010)	−0.034, 0.004	-	0.117
PAI-1 (mg/dL)	Crude	−0.019 (0.022)	−0.063, 0.025	0.401	-
	Model 1	−0.012 (0.033)	−0.077, 0.053	-	0.705
	Model 2	−0.005 (0.033)	−0.071, 0.061	-	0.883
MCP1 (mg/dL)	Crude	−0.047 (0.052)	−0. 15, 0.055	0.363	-
	Model 1	−0.041 (0.072)	−0.184, 0.102	-	0.570
	Model 2	−0.039 (0.073)	−0.184, 0.107	-	
TGF (mg/dL)	Crude	0.034 (0.036)	−0.038, 0.106	0.106	
	Model 1	0.092 (0.038)	0.016, 0.167	**-**	**0.018**
	Model 2	0.101 (0.040)	0.023, 0.180	-	**0.012**
IL-1 β (mg/dL)	Crude	0.001 (0.001)	−0.001, 0.003	0.250	-
	Model 1	0.001 (0.001)	−0.002, 0.003	-	0.596
	Model2	0.001 (0.001)	−0.001, 0.003	-	**0.060**
AIP (mg/dL)	Crude	2.472 (0.001)	0.001, 0.01	0.853	-
	Model 1	0.001 (0.001)	0.001, 0.001	-	0.482
	Model2	0.001 (0.001)	−0.001, 0.011	-	0.072
CRI-I	Crude	0.001 (0.001)	−0.001, 0.002	0.476	-
	Model 1	0.001 (0.001)	−0.001, 0.002	-	0.277
	Model2	0.001 (0.001)	−0.001, 0.002	-	**0.064**
CRI-II	Crude	−3.679 (0.001)	−0.001, 0.001	0.907	-
	Model 1	0.001 (0.001)	−0.001, 0.001	-	0.574
	Model2	0.001 (0.001)	0.001, 0.001	-	0.431
AC	Crude	0.001 (0.001)	−0.001, 0.002	0.476	-
	Model 1	0.001 (0.001)	−0.001, 0.002	-	0.277
	Model2	0.011 (0.001)	0.001, 0.032	-	**0.034**
CHOlIndex	Crude	−0.005 (0.012)	−0.029, 0.019	0.688	-
	Model 1	0.004 (0.015)	−0.026, 0.033	-	0.811
	Model2	0.009 (0.015)	−0.021, 0.040	-	0.547
TyG	Crude	−6.963 (0.001)	−0.001, 0.001	0.794	-
	Model 1	0.001 (0.001)	0.001, 0.001	-	0.610
	Model2	0.001 (0.001)	0.001, 0.001	-	0.501
TyG-BMI	Crude	0.010 (0.023)	−0.035, 0.055	0.648	-
	Model 1	−0.012 (0.028)	−0.67, 0.044	-	0.685
	Model 2	−0.003 (0.029)	−0.060, 0.054	-	0.917
TyG-WC	Crude	0.061 (0.105)	−0.146, 0.267	0.563	-
	Model 1	−0.056 (0.177)	−0.408, 0.296	-	0.752
	Model 2	0.048 (0.188)	−0.324, 0.421	-	0.797

AC, atherogenic coefficient; BFM, body fat mass; BF, body fat; FFM, fat-free mass; FMI, fat mass index; FFMI, fat-free mass index; IL-1β, interleukin-1 beta; MCP-1, monocyte chemoattractant protein-1; SBP, systolic blood pressure; DBP, diastolic blood pressure; FBS, fasting blood sugar; TG, triglyceride; HDL, high-density lipoprotein; LDL, low-density lipoprotein; GPT, glutamic-pyruvic transaminase; GOT, glutamic-oxaloacetic transaminase; PAI-1, plasminogen activator inhibitor- 1, SD standard deviation; hs-CRP, high sensitive- C reactive protein; TF, trunk Fat; VFA, visceral fat area; VFL, visceral fat level; TC, total cholesterol; AIP, Atherogenic index of plasma; TyG, Triglyceride-glucose; TGF, Transforming growth factor; CRI: Cardiac risk index.

Model 1: Adjusted for age, BMI, physical activity, total energy intake, supplements intake, and job status (BMI considered as a collinear variable).

Model 2: In addition controlled for the role of vegetables and legumes.

^*^A P-value obtained from adjustment. All of the p-values obtained from the analysis of the linear regression.

A P < 0.05 was considered significant and p-values equal to 0.05, 0.06, and 0.07 were considered marginally significant.

## Discussion

To the best of our knowledge, this is the first study investigating the relationship between UPF intake and cardiometabolic risk in overweight and obese Iranian women.

**Figure 1:**
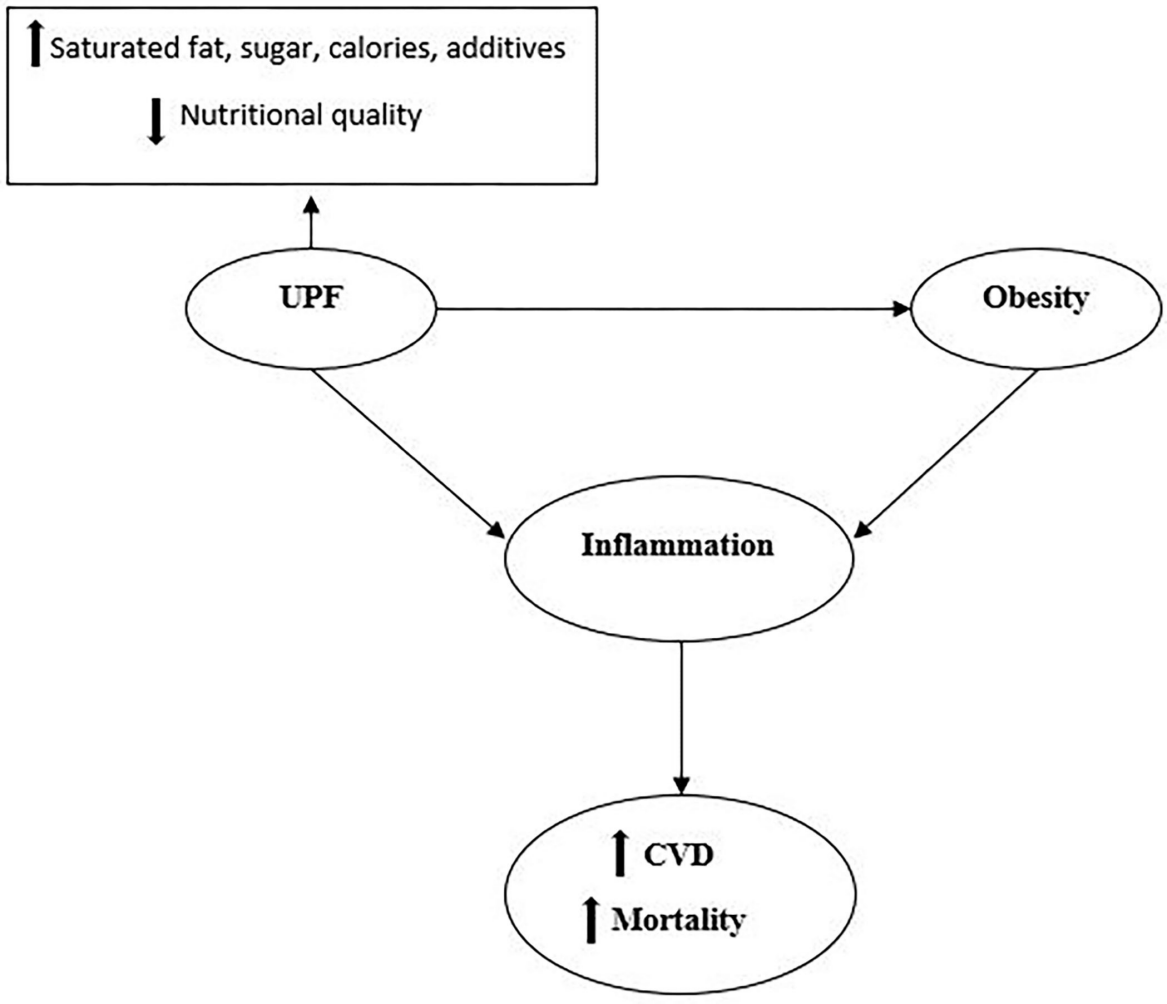
UPFs have higher levels of saturated fats, sugar, salt, additives, calories, and lower nutritional quality. Consumption of UPFs is suggested to have associations with obesity. Both obesity and consumption of UPFs could stimulate the whole chronic inflammation cascade and enhance the risk of CVD and all-cause mortality.

In the current study, we found an inverse association between the NOVA score and FFM. In addition, we observed a positive association between the NOVA score and VFL, AC, the HOMA-IR-index, QUICKI, TC, TGF, IL-1B, and the CRI-I levels. In other words, participants who had higher NOVA scores and consumed higher amounts of UPF had higher levels of VFL, AC, the HOMA-IR-index, QUICKI, TC, TGF, IL-1B, and CRI-I. The positive association observed between UPFs and mentioned markers might be partly explained by their poorer nutritional quality compared with the NOVA scores' lower tertiles. In fact, UPFs tend to be higher in saturated fats, sugar, and energy, and poorer in dietary fiber (5, 9, 21, 33). The positive association between consumption of UPF and inflammatory markers that have been seen among women may be explained by the greater accumulation of body fat in women (34). In line with our study, in 2021, a systematic review and meta-analysis of 7 cohort studies (207,291 adults) showed a significant positive association between UPF consumption and the risk of CVDs among adults with a BMI of more than 25 kg/m^2^ (35). Moreover, a recent narrative review study by Matos et al. (36) concluded that the consumption of UPFs is positively associated with the prevalence of chronic complications, including obesity, hypertension, CVDs, type 2 diabetes, and consequently the risk of all-cause mortality (36). The mechanism by which UPF is associated with CVD is summarized in Figure 1.

In our study, individuals at higher tertiles of NOVA (compared to tertile 1) had higher NC, AC, TyG-WC, HOMA-IR-INDEX, CRI-I, TGF, and hs-CRP levels. Beslay et al., in a large observational prospective study of 110,260 adults, indicated that higher consumption of UPF was associated with a gain in BMI and higher risks of overweight and obesity (37). Also, a prospective cohort of healthy subjects in Italy showed that adults in the highest quartile of UPF consumption had a higher risk of CVD (38). It is well known that adipose tissue produces cytokines that induce inflammatory markers production (39). Thus, the association between the consumption of UPFs and the inflammatory response is expected to be mostly dependent on adiposity. A cross-sectional study displayed that there might be a direct association between consumption of ultra-processed foods and CRP levels, under the assumption that the high-glycemic index of these food products could stimulate the whole chronic inflammation cascade, along with an indirect association mediated by obesity. They suggest that decreased consumption of UPFs can reduce chronic low-grade inflammation, perhaps by reducing obesity (40).

In the present study, participants with higher NOVA scores had higher consumption of cakes and sweets, processed meats, and fast foods. Bonaccio et al., in 2021, indicated that a high proportion of UPF in the diet was associated with an increased risk of CVD and all-cause mortality, probably because of its high dietary content of sugar (38). Rising evidence suggests that the consumption of UPF products determined by the low-nutritional quality and high-calorie content adversely contribute to an unhealthy dietary pattern, which enhances the risk of all-cause mortality as a substantial risk factor (36). In addition, additives in such foods containing noncaloric artificial sweeteners, emulsifiers, and thickening agents cause numerous chronic disorders such as metabolic dysfunction, systemic inflammation, endothelial dysfunction, and disrupted immune response (41–43). More than that, synthetic compounds used in the packaging of UPFs, such as bisphenol A, can act as xenohormones. Particularly, bisphenol A has been indicated to impair reproductive function and augment the risk of cancer-cause mortality (44, 45). Recently, a study conducted in the US displayed that UPF consumption was related to increased exposure to phthalates (44), which has suggested associations with obesity (46). Some food additives specific to UPFs might be involved in obesity etiology. For instance, saccharin, an artificial sweetener, could potentiate glucose-stimulated insulin release from isolated pancreatic β-cells (47), leading to insulin resistance and possibly weight gain. Several emulsifiers (such as carboxymethyl cellulose and polysorbate-80) induced metabolic perturbations, alterations to the gut microbiota, and low-grade inflammation in mice (48). Carrageenan, in the top 20 used additives, might augment insulin resistance and inhibit insulin signaling in mouse liver and human HepG2 cells (49, 50), which might, in turn, induce weight gain (51). Trans fatty acids found in UPFs containing hydrogenated oils have been associated with cardiovascular disease (52) and obesity (53), apparently by altering nutrient handling in the liver, the adipose tissues, and the skeletal muscle (54). Acrylamide, a neo-formed compound created during thermal processing of food as a result of the Maillard reaction, was shown to induce adipocyte differentiation and obesity in mice (55).

The present study possesses some strengths and limitations. At first, to the best of our knowledge, this is the first study to have evaluated the association between processed food intake and CVD risk in overweight and obese Iranian women. Second, dietary intake was assessed using a validated questionnaire. Third, in the current study, we assessed several inflammatory markers, other biochemical parameters, and body composition as risk factors for CVD.

Nevertheless, despite these strengths, we must acknowledge some limitations in the present study. First, the cross-sectional nature of this study limited the ability to suggest a causal relationship between UPF intake and the risk of cardiovascular diseases. Second, some errors may be present in the dietary assessment, mostly due to recall bias and misclassification errors; to overcome such errors, we evaluated biomarkers such as vitamin C to better capture individuals' variability in intakes. Third, our result may not be generalizable to normal-weight women. At final, although we considered known potential confounders, residual confounding cannot be ruled out.

## Conclusion

In conclusion, an increase of one gram of UPFs consumption is associated with an increase in TGF, AC, and VFL but with a decrease in QUICKI. Higher consumption of UPF is significantly associated with an enhanced risk of adult inflammation and cardiometabolic risk factors. Further large studies involving participants of different ages and genders are highly warranted, in addition to experimental studies, to draw a more definite conclusion and disentangle the mechanisms by which UPFs may affect health.

## Data availability statement

Participants of this study disagreed on their data being shared publicly, so supporting data is not available. Further inquiries can be directed to the corresponding author KM, mirzaei_kh@tums.ac.ir; mirzaei_kh@sina.tums.ac.ir.

## Ethics statement

The studies involving human participants were reviewed and approved by Tehran University of Medical Sciences, Tehran, Iran. The patients/participants provided their written informed consent to participate in this study.

## Author contributions

DH and SN wrote the paper. FS and FM-E performed the statistical analyses. SJ, MD, AS, and JB critically reviewed and revised the manuscript. KM had full access to all of the data in the study and took responsibility for the integrity and accuracy of the data. All authors read and approved the final manuscript.

## Funding

This study is funded by grant from the Tehran University of Medical Sciences (TUMS) (Grant ID: 97-03-161-41017).

## Conflict of interest

The authors declare that the research was conducted in the absence of any commercial or financial relationships that could be construed as a potential conflict of interest.

## Publisher's note

All claims expressed in this article are solely those of the authors and do not necessarily represent those of their affiliated organizations, or those of the publisher, the editors and the reviewers. Any product that may be evaluated in this article, or claim that may be made by its manufacturer, is not guaranteed or endorsed by the publisher.
